# Vehicle Destination Prediction Using Bidirectional LSTM with Attention Mechanism

**DOI:** 10.3390/s21248443

**Published:** 2021-12-17

**Authors:** Pietro Casabianca, Yu Zhang, Miguel Martínez-García, Jiafu Wan

**Affiliations:** 1 Department of Aeronautical and Automotive Engineering, Loughborough University, Loughborough LE11 3TU, UK; P.Casabianca@lboro.ac.uk (P.C.); M.Martinez-Garcia@lboro.ac.uk (M.M.-G.); 2 School of Mechanical and Automotive Engineering, South China University of Technology, Guangzhou 510641, China; mejwan@scut.edu.cn

**Keywords:** attention mechanism, bidirectional long short-term memory, deep learning, vehicle destination prediction

## Abstract

Satellite navigation has become ubiquitous to plan and track travelling. Having access to a vehicle’s position enables the prediction of its destination. This opens the possibility to various benefits, such as early warnings of potential hazards, route diversions to pass traffic congestion, and optimizing fuel consumption for hybrid vehicles. Thus, reliably predicting destinations can bring benefits to the transportation industry. This paper investigates using deep learning methods for predicting a vehicle’s destination based on its journey history. With this aim, Dense Neural Networks (DNNs), Long Short-Term Memory (LSTM) networks, Bidirectional LSTM (BiLSTM), and networks with and without attention mechanisms are tested. Especially, LSTM and BiLSTM models with attention mechanism are commonly used for natural language processing and text-classification-related applications. On the other hand, this paper demonstrates the viability of these techniques in the automotive and associated industrial domain, aimed at generating industrial impact. The results of using satellite navigation data show that the BiLSTM with an attention mechanism exhibits better prediction performance destination, achieving an average accuracy of 96% against the test set (4% higher than the average accuracy of the standard BiLSTM) and consistently outperforming the other models by maintaining robustness and stability during forecasting.

## 1. Introduction

Satellite navigation has become a vital tool for drivers, with the majority of motorists relying on Global Positioning System (GPS) devices to arrive at their destination [1]. Almost every new vehicle and smart device has access to satellite networks, such as Global Navigation Satellite System (GNSS) and GPS receivers. This enables the possibility of many new and improved location-based services, such as points of interest, estimated time of arrival, live traffic updates, and fastest and alternative routes [2,3].

Having the capability of recording a vehicle’s position also enables predicting a driver’s destination based on their driving schedule and destination patterns. Such systems are increasing in popularity due to the many potential advantages they bring forth, without requiring any explicit input from the driver. One predominant advantage is being able to provide personalized navigational advice and alerts throughout each journey [2].

Such advice may include warning of potential hazards, route changes to miss traffic congested areas [4], highlighting useful stopping places along the route, and finding optimal refueling/recharging stations. Moreover, by knowing the user’s destination, the vehicle can optimize its energy consumption. This is especially useful with hybrid and electric vehicles, since they may automatically discharge batteries if a recharging station is available nearby, to improve battery charging cycles. Research has shown that with prior destination knowledge hybrid fuel economy can be improved by as much as 7.8% [5].

Such systems are useful in large cities that are moving toward greener energy alternatives, aimed at reducing emissions. For example, London has ultralow emission zones that were expanded from central London up to the north and south circular roads after 25 October 2021 [6]. Additionally, London has begun introducing zero emission zones to reach their City Corporation’s draft transport strategy targets [7]. If a driver’s destination is known to be within these zones, a hybrid vehicle travelling from outside of London can automatically optimize fuel usage when outside, saving its battery energy for when travelling within these low emission zones.

Though it is true that a driver could be asked to input a destination prior to departure, it is more convenient that they will not be required to input this information, especially for their frequently travelled routes [8].

This paper develops a reliable method for destination prediction using deep learning models, which includes the investigation of Long Short-Term Memory (LSTM) networks with the addition of an attention mechanism. Accordingly, the main contributions of this paper follow:

- Explore the viability of advanced deep learning methods in the automotive and associated industrial domain;
- Develop a reliable vehicle destination prediction method using the GeoLife GPS Trajectory [9,10,11] dataset;
- Study LSTM networks compared to conventional neural networks;
- Investigate the effect of adding bidirectionality to an LSTM network on the robustness of the destination predictions.
- Include novel techniques such as attention mechanisms to further improve network performance.

The rest of the paper is organized as follows. Section 2 reviews the state-of-the-art related to this research. Section 3 describes the data sources and the data preparation, while Section 4 provides details of the applied deep learning architectures. Section 5 presents the analysis results and comparisons, and, finally, Section 6 concludes the paper.

## 2. Related Work

In the field of route and destination prediction, a substantial amount of research has been conducted—testing different types of techniques and systems to improve predictions [12]. These techniques are simplified to two categories, route matching algorithms [3,13,14,15] and probabilistic modelling systems [2,12,16,17,18,19]. Froehlich et al. [3] introduced an algorithm that matches the current route to a past route by using a similarity score. The route with the highest similarity score would then be the predicted route. On the other hand, common probabilistic methods include the use of a Markov chain model and its Hidden Markov Model (HMM) variant [12,19]. Markov processes are essentially without memory, as they predict based on a current state. In comparison, there exist explicit memory networks, such as LSTMs, which use a history of past locations, times, and patterns to forecast the future.

For any data-driven model, data preparation has a significant effect on the model performance; related work inputs require significant effort in cleaning, filtering, and improving the training data [3,20]. Tanaka et al. [20] included common contexts of daily driving with their trajectories, such as the time and day of the week, showing promising results. For destination prediction, the method of clustering destinations has proven to perform well [2,16,17]. Destination clustering involves grouping destinations that are relatively close to each other as they likely pertain to the same destination since a driver will not park in the same spot every time. This also converts the destinations from points with longitude and latitude positions to a single integer, thus allowing for destination classification.

State-of-the-art methods for predicting vehicle destinations include random forests and deep neural networks [2,17,19]. Random forests have been used in destination prediction problems with relative success [2,19]. While they tend to train faster and require less computational power than deep learning models [21], their weakness becomes apparent as a user completes more journeys. To train and retrain a random forest model, all of the training data must be present in system memory. Thus, as more journeys are completed, more storage and computational power are required. In contrast, deep learning models can process the training data in batches, and sometimes forget previous training samples. Their weights can be updated with new journeys, allowing the system to automatically delete previous ones and save storage. Such a setup would also reduce the required computational power compared to having to train a model from scratch every time with an endlessly increasing dataset size. Additionally, drivers may change their habits through time. Thus, deep neural networks will better adapt to these changes by emphasizing its training more on recently recorded travel paths.

In recent years, the utilization of deep learning methods for any type of time series forecasting has greatly expanded since the introduction of Recurrent Neural Networks (RNNs), including Gated Recurrent Units (GRUs) and the LSTM model [19]. RNNs have demonstrated superior results in sequential time series learning tasks due to their explicit memory tracking, providing context on the previous states of a time series [22]. However, basic RNNs are limited in dealing with longer sequence data due to the exploding and gradient vanishing phenomena [17,23]. The introduction of gate structures, found in LSTMs, has shown to partially mitigate these problems, and has become widely adopted in both trajectory [24,25,26] and destination prediction problems [17,19,27,28,29], achieving promising outcomes. Brébisson et al. [17] concluded that using a Bidirectional LSTM (BiLSTM) improved prediction performance as compared to unidirectional LSTM, due to the inputs being accessed in both directions. The benefit of this setup compared to a unidirectional LSTM is that the output of the BiLSTM holds information about the past and future states, whereas the unidirectional LSTM only preserves information of the past [17].

LSTMs have, however, shown to suffer from a constraint originating from their encoder–decoder architecture; all their input sequences are forcibly encoded into a fixed-length vector representation [30], which is believed to limit the LSTMs performance, especially with long input sequences [31]. By introducing an attention mechanism over the output of the LSTM network, the encoder–decoder architecture is freed from the fixed-length vector representation, leading to a higher performing model [31]. Attention mechanisms have recently demonstrated success in many tasks due to their ability to selectively focus on and extract the important features of the input data, thus improving model performance [32,33]. However, these tasks are mainly related to natural language processing and text-classification-related applications. Hence, this paper investigates the viability of these techniques in the industrial domain. Specifically, it uses attention mechanisms applied to the outputs of LSTM networks to conclude if this improves model performance from the standard LSTM when used for destination prediction.

## 3. Data Preparation

**Figure 1:**
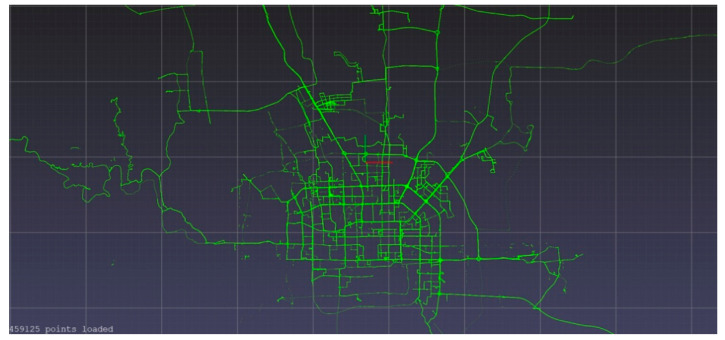
Car trajectories around the capitol of China, Beijing.

In this paper, the GeoLife GPS Trajectories [9,10,11] dataset is used. While this dataset contains trajectory information for many different forms of transportation (e.g., walking, flying, boating, taxiing), mainly around the city of Beijing, China, only the car trajectories were used (depicted in Figure 1). The raw datafile has multidimensional information, such as date and time, latitude and longitude positions, and user number. The first preprocessing step was to separate distinct journeys by using the time stamps. The raw datafile contains assembled ordered trajectories, and as the “trajectories were recorded in a dense representation of every 1–5 s”, the end of one journey is determined when the following timestamp occurred more than 300 s later. In other words, if data are not collected for more than 5 min, it is deemed a sufficiently long stoppage time for considering it a new journey.

Here, any journey that lasts less than 10 min is discarded due to it having negligible information. The destinations of each remaining journey are then converted from longitude and latitude locations to clusters, as in [2,16,17]. This is done since it is unlikely that a driver always parks in the same location when reaching the same destination. Instead, they may park within a certain radius. In this case, the radius was set to 500 m empirically. Having destinations as clusters makes the destination prediction task a classification problem.

**Figure 2:**
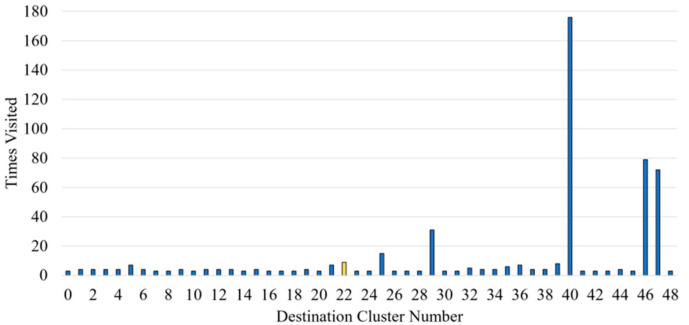
Number of journeys reaching each specific destination cluster. A destination where one journey was removed prior to model training (yellow) is used as an unseen full journey test.

To allow for the predictability of a destination, any cluster that is only visited once or twice is removed, eventually reducing the number of possible destinations to 49 clusters. Figure 2 shows the data split with the number of times a destination is visited. D40 (D stands for Destination) was the most frequent, followed by D46 and D47, which have in comparison almost 100 less visits each.

One full journey, leading to D22, is arbitrarily removed from the dataset prior to the train–validation–test split for model training and testing. This allows the models to be tested independently on a whole journey that is not part of the training process. This destination is not visited frequently, which can demonstrate how the model performs against a journey unlikely to have been seen previously. Additionally, several frequent and infrequent journeys are used to test the models on routes they have seen. Test journeys are used to demonstrate the performance of a model for an entire journey, as opposed to its performance against a randomized test set of shuffled points from different destinations.

The final dataset is organized with feature inputs, including (1) current latitude, (2) current longitude, (3) user ID, (4) time, (5) date, (6) day of the week, (7) change of distance in latitude, (8) change of distance in longitude, and the target output as (9), the destination cluster number.

## 4. Methodology

The methodology is implemented using Python 3.6. The packages utilized include scikit-learn 0.24.2 [34], TensorFlow 2.6.0 [35], and Attention 4.0 [36]. Scikit-learn is used to split the training dataset into training, validation, and test sets. TensorFlow is used to build and train the deep neural networks, and the Attention package is used to include the attention mechanism in the models.

### 4.1. Static and Dynamic Dataset Setup

Prior to model training, the data are organized into two arrays. The first array contains the features and the second contains the targets, i.e., destinations. The static setup simply relates one row of features to one destination. However, the dynamic setup contains a third dimension, related to time. A moving window is applied to the timesteps by connecting twenty rows of features to one output destination. In theory, changing the data setup from static to dynamic should improve performance since the model will be given more information, such as the direction of travel. Brébisson et al. [17] input five successive GPS points as the dynamic setting, however, after empirical experimenting, here it is found that inputting twenty rows of features shows better results while still being an acceptable amount of time to wait before the models start predicting. With the dataset used, GPS points are every 1–5 s, indicating that the longest time before the models can start predicting is 100 s into a journey.

### 4.2. Model Training

Before training, the dataset is split into three subsets: training, validation, and testing. The testing set is only used at the end of training, whereas the training and validation sets are used throughout training.

**Figure 3:**
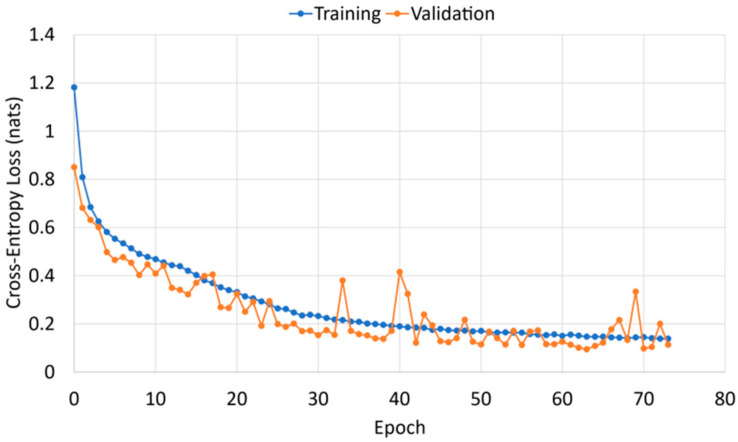
Training curve of a BiLSTM with an attention mechanism. The lowest validation loss occurs at epoch 63 and this model is saved.

Through empirical tuning, the optimizer “Adam” is used with a learning rate set to 0.001, and a small batch size of 32 is used. To limit the likelihood of a model overfitting to the training dataset, early stopping and model checkpoints are used to save the model with the lowest validation loss score. If after 10 consecutive epochs the validation loss score does not reduce, training stops, as shown by the training curve in Figure 3. This loss was calculated by TensorFlow using the loss function sparse categorical cross-entropy. The validation loss score is calculated at the end of each epoch and used to adjust network weights.

### 4.3. Deep Learning Models

To keep model performance comparison consistent and to allow for an adequate analysis of the impact of adding a layer, all the tested models had the same dense layers as the Dense Neural Networks (DNNs), with only an additional layer at the start to implement the LSTM or LSTM with Attention.

#### 4.3.1. Dense Neural Network

As a baseline for comparison, a simple three-layer sequential DNN was optimized and trained with the static and dynamic data setup (Table 1). In artificial neural networks, a dense layer is regarded as a fully connected layer, which performs a linear operation on inputs or the previous layers’ outputs. In static set up, the inputs are an array of features, while in dynamic setup the inputs are multiple rows of features representing a time moving window. This experiment is to confirm that a dynamic setup does improve model performance. It also allows a comparison for when the LSTM and attention layers are added. To allow for the model to accept the dynamic data input, a one-dimensional global average pooling layer is added prior to feeding the inputs to the first dense layer. This is shown by the greyed row in Table 1, which is not present for the static data setup.

DNNs simply work with the neurons in a dense layer receiving as inputs the outputs from all the neurons in the previous dense layer, using matrix-vector multiplications, where the values in the matrix are the parameters which are updated through backpropagation during training. Therefore, DNNs have no memory elements, and each sequential layer only relies on the outputs of the previous layer.

#### 4.3.2. LSTM and Bidirectional LSTM Models

All RNNs have feedback loops in their recurrent layer that allow them to maintain information over time. However, LSTMs include memory cells, which are a set of gates used to control when information is input, output, and forgotten in memory [37]. These memory cells allow information to be maintained in memory for longer periods of time compared to feedback loops.

Thus, LSTMs have become widely adopted in trajectory and destination prediction methods. Since Brébisson et al. [17] found that adding bidirectionality to the LSTM model improved the model’s performance, this paper also aims to investigate if BiLSTM models improve performance for destination prediction.

Conventional LSTM is unidirectional, which only preserves information from the past states. In contrast, BiLSTM is bidirectional, where the information is passed on from both the past and the future states at a point in time. It should be more suited when both past and future contexts are relevant in the prediction task. Table 2 shows the architecture used, where the first layer is changed to a bidirectional LSTM layer.

#### 4.3.3. LSTM with Attention and BiLSTM with Attention

With traditional sequence-to-sequence models, all the intermediate steps of the encoder are disregarded and only the final states, summarized into a vector, are used to initialize the decoder. While this method works for small sequences, when using larger sequences, the single vector bottlenecks model performance. The main benefit of attention mechanisms is that the intermediate encoder states can be used, providing improved context vectors for the decoder [31].

A many-to-one attention mechanism for Keras is used [36]. The context vectors are calculated by the multiplication of the encoder hidden states and their attention weights, where the attention weights are obtained by performing the softmax operation on the alignment scores of the hidden states. This attention mechanism is applied to the outputs of the LSTM layer (Table 3), taking in a three-dimensional tensor with shape (batch size, timesteps, input dimension), and returning a two-dimensional tensor with shape (batch size, 128). The attention mechanism is applied to both the LSTM and BiLSTM models to investigate the model performance.

## 5. Results

To evaluate the performance of each model, two test scenarios were carried out. One involves using the test set taken from the train–validation–test split at the start of model training, which is made up of many different journey destinations shuffled. The other scenario uses a full unseen journey, which is manually removed from the training dataset prior to model training, to compare when each model starts to predict the correct destination. In addition, seen journeys are also used for checking the models against past journeys.

### 5.1. Model Performance against Test Set

**Figure 4:**
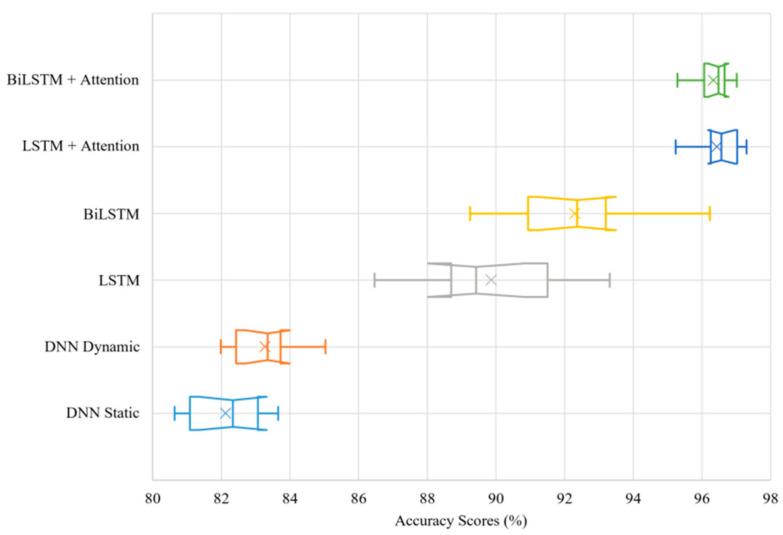
Model accuracy evaluations for ten training and testing runs shown by boxplots and × indicating the mean.

Figure 4 shows the accuracy scores for all the models studied (Section 4.3), and the average performance scores are summarized in Table 4. In Figure 4, each boxplot corresponds to the results obtained from the model indicated in the Y-axis. The X-axis represents the accuracy in percentage. Both mean and median are demonstrated in the boxplots. The length of the whiskers is computed by using the lowest and highest model performance results. The notches represent the 95% confidence intervals. Each model result includes ten individual training–testing runs. The exclusive method has been used for the calculation of the interquartile range.

A notable performance improvement is shown between the static and dynamic DNN models. Since the two DNN models are identical, with the only difference being the addition of a one-dimensional global average pooling operation to allow training with the dynamic data, the results show that a slight performance improvement is already achieved by converting the training data from a static to a dynamic setup. Confidence intervals overlapping indicate that the performance improvement is not statistically significant to conclude if one model outperforms the other entirely. However, because there is not a complete overlap between the two models, it is suggested that the dynamic DNN outperforms the static DNN.

On the other hand, it can be confidently claimed that the LSTM models show statistically significant performance improvements over the DNNs. Adding an LSTM layer, as hypothesized, significantly improves model performance. Although changing this to a BiLSTM layer shows a further increase in performance, confirming the findings from Brébisson et al. [17], the confidence intervals of the two boxplots only slightly overlap. Therefore, although it cannot be statistically concluded that BiLSTM outperforms LSTM, it can be stated with sufficient certainty that introducing bidirectionality improves destination prediction performance since the confidence interval overlapping is minimal. However, this can be confirmed by the full journey tests to be discussed in Section 5.2.

The LSTM and BiLSTM models produce results with a larger interquartile range compared to the DNN models, which means their performance results are more variable, but nevertheless superior. The larger variability in performance is likely caused by the model’s constraint of having its input sequences forcibly encoded into a fixed-length vector representation [30], resulting in lower performance for the runs in which the constraint limits training.

Introducing an attention mechanism over the output of the LSTM network should free the architecture from the constraining fixed-length vector representation, leading to higher performance. When applied, the attention layer results in a significant performance improvement to both the LSTM and BiLSTM models (with no confidence interval overlap), while also significantly reducing the interquartile range, showing even more stability in performance scores than the DNN models. Therefore, attention mechanisms applied to the outputs of LSTM networks do improve model performance from the standard LSTM when used for destination prediction.

In a similar way, the confidence intervals overlap between the BiLSTM with Attention and the LSTM with Attention results significantly. Therefore, even though the LSTM with Attention achieves a slightly higher peak in accuracy score, the difference between the overall performance of the two models is not statistically significant.

Training time is another important factor to consider, as more complex models may not be viable if their training times are too long, even if they have improved performance scores. Python codes are executed using Google Colaboratory Pro+ [38] with specifications: 2 Intel(R) Xeon(R) CPU @ 2.20 GHz, 12 GB RAM, 180 GB HDD available. Table 5 summarizes the average time to train for each model used in this paper. While there is a significant increase in training time between the DNN and LSTM models, it is justifiable due to the major gain in performance score—with an average accuracy improvement of about 6.6% from the dynamic DNN to the LSTM model. Remarkably, Table 5 also shows that the addition of the attention mechanism leads to a lower averaged training time compared to the exact same model without the attention layer (for example, LSTM vs. LSTM + Attention). The attention models also have the highest performance, improving from the DNN models by more than 13% and achieving a very high average accuracy of more than 96%, further justifying the increased training time.

### 5.2. Full Journey Tests

Although the LSTM with Attention model shows the highest average accuracy with shorter training time, it cannot be confirmed as the best model due to the performance difference being not statistically significant against the BiLSTM with Attention model. Therefore, the models are tested for entire journeys. Factors such as how quickly a model starts predicting the correct destination, and how stable the model is with its predictions are important. Additionally, it is useful to deduce why a model may predict a wrong destination to ensure, at least, that the incorrect prediction could still be a plausible destination based on the part of the route the vehicle is on.

The dense models are omitted from the full journey tests for simplicity since they have been significantly outperformed against the previous testing dataset.

One unseen journey is used for testing, as explained in Section 3. Frequently and infrequently seen journeys are also used to test the performance of the models against journeys it has seen in the past—as would more likely be the case in real-world applications.

#### 5.2.1. Frequent Destination Tests

For this test, the models are fed one journey from each destination that is visited more frequently (as visualized from Figure 2). Since these destinations are visited much more than the other destinations in the training dataset, it should be straightforward for all the models to predict the destination quickly and confidently.

Table 6 details the percentage of journey that is correctly predicted by each model for the selected frequent journey. These tests confirm that all four models are working extremely reliably when tested against frequent destinations, making them successful for the average commuter. Since there is little performance separation between these models based on these results, the next sections explore the models’ performance on three more infrequent destinations, one of which is unseen and extracted from the dataset prior to training.

#### 5.2.2. Unseen Journey with D22

**Figure 5:**
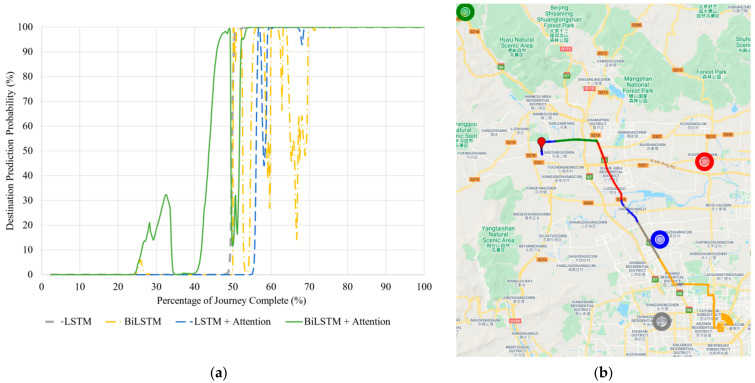
(**a**) Correct destination prediction versus percentage complete for the unseen journey to Destination 22. (**b**) BiLSTM with Attention destination predictions visualized along the same route. The red pin is the starting point. Map data © 2021.

Figure 5 shows how each model’s prediction probability changes for the unseen journey to D22.

The LSTM model is the most stable once it predicts the correct destination around 50% of the journey, remaining fixed with that prediction for the remainder of the travel. The BiLSTM also predicts the correct destination around the same time; however, its predictions are much more unstable, as is shown by the yellow curve oscillating past the 50% probability mark until it stabilizes at 70% completion of the journey (Figure 5a). The LSTM with Attention model is the last to predict the correct destination; however, it is much more stable than the BiLSTM model, predicting the incorrect destination for only three timesteps after it predicts the correct destination, and remaining stable for the remainder of the journey. Noticeably, the BiLSTM with Attention model shows the most reliability, predicting D22 around 5% of the journey earlier than the LSTM model and only losing prediction accuracy for a small section of the journey around the 50% journey mark. Better performance is also reinforced by Table 7, showing that the BiLSTM with Attention predicts the most percentage of the journey with correct destination.

Although the BiLSTM with Attention performed the best for this test, it is important to check that the incorrect predictions it makes are logical. In other words, it should be assured that the model is not predicting a destination that is unreachable from the road in which the car is travelling. Such a prediction would indicate a problem with the trained model. This investigation is done as shown in Figure 5b, which includes the whole test journey to D22. The starting point is visualized by the red pin and the correct destination is shown by the orange circle. The different coloured sections along the route indicate what destination the BiLSTM with Attention model is predicting at that position. Each route colour fits with its respective destination colour (these are previously clustered destinations). The destination cluster area is shown by the transparent circles, whereas the solid outer circles are for easier visualization of their locations.

Predicting a destination from a journey is difficult due to the many routes that could be taken to reach the same, or even slightly different, destination. Figure 5b illustrates this on a few occasions. For example, at the junction highlighted by J1, if the car had turned to the left, it would be on a direct route to the green destination. However, as soon as the vehicle makes a right turn, the model recognizes that the driver is not heading toward the green destination and predicts red instead, which is still reachable by different road intersections. These predictions, while incorrect, are reasonable.

As the vehicle continues along the route, predictions change as roads that lead to the incorrect destinations are passed. This is credible because it shows that the destinations the BiLSTM with Attention model is predicting are logical.

Another interesting prediction change occurs straight after a highway intersection, highlighted by J2. The model has started predicting the correct destination, until it changes back to grey for a few timesteps. This means that this section of the journey is likely very similar between the grey and the orange destination, causing uncertainties of the model in its prediction. Nonetheless, once the car’s position is updated past the highway junction, the model predicts the correct destination for the remainder of the test journey.

Against a journey which is not part of the training dataset, the model showed successful generalization and made logical predictions throughout the unseen test journey.

#### 5.2.3. Seen Journey with D5

**Figure 6:**
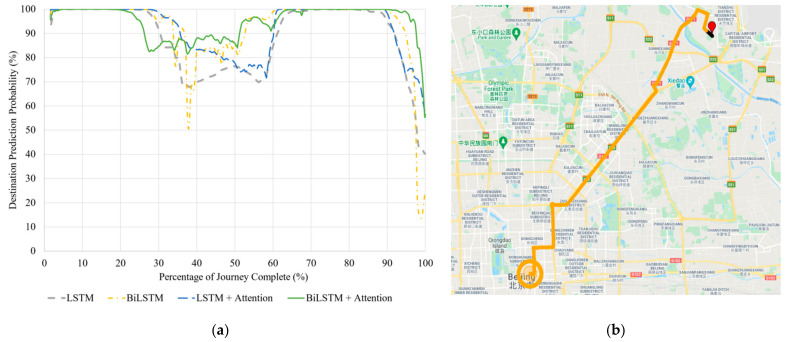
(**a**) Correct destination prediction certainty versus percentage complete for the seen journey to Destination 5. (**b**) BiLSTM with Attention destination predictions visualized along the same route. The red pin is the starting point. Map data © 2021.

Figure 6 shows how each model’s prediction probability changes for the seen journey to D5.

All models perform perfectly with this seen journey (Table 8), with only the LSTM and BiLSTM models giving incorrect predictions at the very end of the journey, as shown by the rapid drop in probability from 90% journey onwards in Figure 6a. This drop occurs for all tested models; however, the models with attention mechanism have probabilities that remain above 50%. Throughout the journey, the models with attention mechanism show higher probability levels for most of the time, indicating that they have more stability in their predictions.

Figure 6b shows the entire journey, with the initial black points of output being the first 20 timesteps that the model cannot make predictions on due to the moving time window. On the 21st timestep, the BiLSTM with Attention model immediately predicts the correct destination and remains with this prediction for the entire journey. The drop in probability by the end of the journey indicates that the vehicle is starting to head on routes leading to other destinations.

#### 5.2.4. Seen Journey with D15

**Figure 7:**
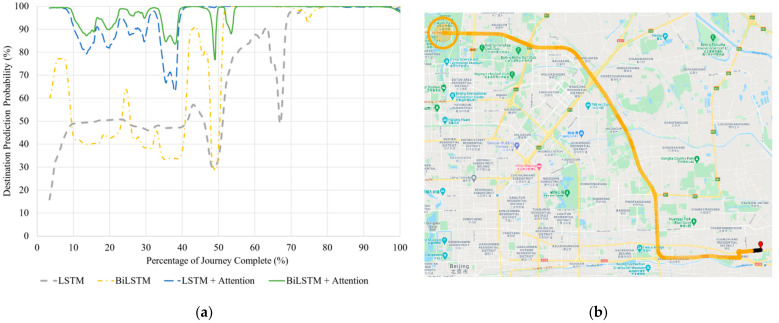
(**a**) Correct destination prediction certainty versus percentage complete for the seen journey to Destination 15. (**b**) BiLSTM with Attention destination predictions visualized along the same route. The red pin is the starting point. Map data © 2021.

Figure 7 shows how each model’s prediction probability changes for the seen journey to D15.

In this test, as detailed in Table 9, the models with attention mechanism significantly outperform the models without it. Figure 7a,b shows that all the models achieve 100% probability once the vehicle passes the first two highway intersections and is on a direct route to the destination. However, the models without attention have relatively low prediction probabilities for the first half of the journey, whilst the models with attention mechanism maintain more stable and high prediction probabilities throughout, stabilizing close to 100% probability after the mid-journey mark.

Based on all the results of each test, the BiLSTM with Attention model consistently shows the highest performance against the test set, demonstrating high accuracy and prediction stability against the test journeys. Although the BiLSTM achieves higher performance scores than the LSTM model against the shuffled test set, the independent journey tests show that their performance is very similar. While there is no significant performance difference between the two models with the attention mechanism against the shuffled test set (Figure 4), the higher certainty, prediction stability, and the earlier correct destination prediction from the unseen journey test (Figure 5a and Table 7) suffice to demonstrate that the BiLSTM with Attention model is most adequate in this case for destination prediction.

## 6. Conclusions

This paper presented a reliable methodology for vehicle destination prediction. It demonstrated the effectiveness of applying an attention mechanism over the output of a BiLSTM network for predicting the destination of a vehicle based on its journey history, achieving classification accuracy higher than 96% from the test set, based on the GeoLife GPS Trajectory [9,10,11] dataset.

While the models with attention have outperformed the standard LSTM and BiLSTM models, the performance difference against the test set between the two models with attention is found not statistically significant. However, further investigating the models against test journeys provided more insights. Against the unseen test journey, consistently outperforming the other models, earlier prediction, and maintaining strong prediction stability throughout the journey, the BiLSTM with Attention is proved to be the most adequate model in this case for predicting vehicle destinations. The performance improvement shown by attention makes sense because attention mechanisms work better with larger sequences as they use intermediate encoder states as the inputs into the decoder, as opposed to traditional sequence-to-sequence models that can only store the final encoder states in a single vector, causing performance bottlenecks when sequences become larger [31].

Future work could involve testing these models in a real-world scenario with a dataset that continuously expands as a user completes more trips, to achieve a more in-depth study on how the models perform when there are less or more data available. Additionally, it will be useful to introduce an uncertainty measure, e.g., entropy, to the predicted probabilities of the destinations [2]. This will help identify the reliability of the prediction model, which will then account for the cases that comprise more than one common destination.

## Figures and Tables

**Table 1:** DNN model architecture.

Layer Type	# of Neurons		Activation
Global Average Pooling 1D *	-	-	-
Dense	256	-	ReLU
Dropout rate	-	0.1	-
Dense	128	-	ReLU
Dropout rate	-	0.1	-
Dense (Output)	49	-	Softmax

* Grey row is only needed when the dynamic data setup is used.

**Table 2:** LSTM and BiLSTM model architecture.

Layer Type	Memory Units	# of Neurons		Activation
(Bidirectional) LSTM *	32	-	-	-
Global Average Pooling 1D	-	-	-	-
Dense	-	256	-	ReLU
Dropout rate	-	-	0.1	-
Dense	-	128	-	ReLU
Dropout rate	-	-	0.1	-
Dense (Output)	-	49	-	Softmax

* The first layer changes according to whether LSTM is used (not bidirectional) or BiLSTM is used.

**Table 3:** LSTM and BiLSTM with Attention models.

Layer Type	Memory Units	# of Neurons		Activation
(Bidirectional) LSTM *	32	-	-	-
Attention	-	32	-	-
Dense	-	256	-	ReLU
Dropout rate	-	-	0.1	-
Dense	-	128	-	ReLU
Dropout rate	-	-	0.1	-
Dense (Output)	-	49	-	Softmax

* The first layer changes according to whether LSTM is used (no bidirectional) or BiLSTM is used.

**Table 4:** Average performance scores of the models against test set.

	Average Performance Scores (%)			
	Accuracy	Precision	Recall	F-Score
DNN Static	82.127	82.405	82.127	81.935
DNN Dynamic	83.272	83.552	83.272	83.136
LSTM	89.860	90.165	89.860	89.820
BiLSTM	92.291	92.489	92.291	92.274
LSTM + Attention	96.431	96.561	96.431	96.431
BiLSTM + Attention	96.330	96.434	96.330	96.328

**Table 5:** Model average training time from ten runs vs. increase of accuracy.

	Time (hrs:mins:secs)	Increase of Accuracy (%)
DNN Static	00:32:05	baseline
DNN Dynamic	00:21:50	1.145
LSTM	01:36:34	7.733
BiLSTM	01:49:44	10.164
LSTM + Attention	01:31:42	14.304
BiLSTM + Attention	01:49:17	14.203

**Table 6:** Results from testing each model on a frequent destination journey.

	Percentage of Journey Correctly Predicted (%)				
Destination Cluster	25	29	40	46	47
LSTM	100	100	100	100	96.477
BiLSTM	100	98.180	100	100	98.660
LSTM + Attention	100	98.608	100	96.970	94.128
BiLSTM + Attention	100	98.394	100	100	96.980

**Table 7:** Percent of journey correctly predicted for all models tested against the unseen journey to Destination 22.

	Percentage of Journey Correctly Predicted (%)
LSTM	51.590
BiLSTM	46.463
LSTM + Attention	44.756
BiLSTM + Attention	55.370

**Table 8:** Percent of journey correct for all models tested against the seen journey to Destination 5.

	Percentage of Journey Correctly Predicted (%)
LSTM	99.608
BiLSTM	97.416
LSTM + Attention	100
BiLSTM + Attention	100

**Table 9:** Percent of journey correct for all models tested against the seen journey to Destination 15.

	Percentage of Journey Correctly Predicted (%)
LSTM	63.238
BiLSTM	67.285
LSTM + Attention	100
BiLSTM + Attention	100
